# Exploring the genetic variability of the *PRNP* gene at codons 127, 142, 146, 154, 211, 222, and 240 in goats farmed in the Lombardy Region, Italy

**DOI:** 10.1186/s13567-024-01353-3

**Published:** 2024-08-06

**Authors:** Carlotta Ferrari, Chiara Punturiero, Raffaella Milanesi, Andrea Delledonne, Alessandro Bagnato, Maria G. Strillacci

**Affiliations:** Department of Veterinary Medicine and Animal Science, Università degli Studi di Milano, Via dell’Università 6, 26900 Lodi, Italy

**Keywords:** goat, scrapie resistance, *PRNP*, transmissible spongiform encephalopathy, single nucleotide polymorphism

## Abstract

Scrapie is a transmissible spongiform encephalopathy affecting sheep and goats. The prion protein-encoding gene (*PRNP*) plays a crucial role in determining susceptibility and resistance to scrapie. At the European level, surveillance of scrapie is essential to prevent the spread of the disease to livestock. According to the Regulation EU 2020/772 polymorphisms K222, D/S146 could function as resistance alleles in the genetic management of disease prevention. In Italy, a breeding plan for scrapie eradication has not been implemented for goats. However, surveillance plans based on the *PRNP* genotype have been developed as a preventive measure for scrapie. This research aimed to describe the polymorphisms at 7 positions within the *PRNP* gene in 956 goats of the Alpine, Saanen and mixed populations farmed in the Lombardy Region in Italy. *PRNP* polymorphisms were detected using single nucleotide polymorphism markers included in the Neogen GGP Goat 70 k chip. The K222 allele occurred in all populations, with frequencies ranging from 2.1 to 12.7%. No animals carried the S/D146 resistance allele. However, it has been demonstrated that polymorphisms in the other positions analysed could influence resistance or susceptibility to scrapie outbreaks in different ways. Ten potentially distinct haplotypes were found, and the most prevalent of the three populations was H2, which differed from the wild type (H1) in terms of mutation (S vs P) at codon 240. This study provided additional information on the genetic variability of the *PRNP* gene in these populations in the Lombardy region of Italy, contributing to the development of genetic control measures for disease prevention.

**Supplementary Information:**

The online version contains supplementary material available at 10.1186/s13567-024-01353-3.

## Introduction

Prion diseases, also known as transmissible spongiform encephalopathies (TSE), are a group of rare neurodegenerative disorders with relatively long incubation periods and invariably fatal outcomes [1]. They affect both humans and other mammals, including goats and sheep. Well-known variants include bovine spongiform encephalopathy (BSE) in cattle [2], scrapie in sheep and goats [3], chronic wasting disease (CWD) in elk and deer [4], and Creutzfeldt-Jakob disease in humans [5]. The most recently discovered prion disease, Camel Prion Disease (CPD), was identified in dromedary camels in Algeria [6]. The transmission of scrapie primarily occurs through direct or indirect exposure to the placenta or body fluids such as saliva, blood, and urine from diseased animals [7, 8]. Clinical symptoms can include behavioral changes, vision loss, coordination issues, motor imbalance, increased excitability, and tremors [9]. The prion protein, known as the cellular prion protein PrP^C^, can transform into a pathological isoform called PrP^Sc^ (prion protein associated with scrapie), leading to progressive brain damage [10, 11]. PrP^Sc^ has been detected in several tissues, including the nervous system, tonsils, spleen, lymph nodes, nictitating membrane, muscles, placentas, distal ileum, and proximal colon, with direct implications for the spread of the disease within animal populations [9, 12, 13].

Despite differences in species and triggers, prion diseases share common features, such as the generation of spongiform lesions in the central nervous system (CNS) due to neuronal loss and the ability to transmit to healthy individuals under certain circumstances [5, 14]. Currently, two types of scrapie exist: classical and atypical, also known as Nor98 [15]. Both are chronic conditions, but they differ in the location of PrP^Sc^ deposits and lesions. In atypical scrapie, the cerebellum is the primary site of damage in the CNS, while in classical scrapie, it is the medulla oblongata [15].

The surveillance of scrapie is vital for identifying animals that carry PrP^Sc^ or are susceptible to it to prevent the spread of the disease among livestock. Regulation (EC) No. 999/2001 has been a fundamental tool at the European level for TSE prevention, control, and eradication, including scrapie. It has now been amended by Regulation (EU) 2020/772, which introduced rules that limit culling and destruction to animals susceptible to this disease. This implies that if animals in the flock carry alleles conferring genetic resistance to classical scrapie (e.g., K222 or S/D146 alleles), they are not subjected to culling and destruction measures, thereby contributing to a more targeted management approach based on genotype. In particular, the European Food Safety Authority (EFSA) plays a crucial role in the assessment and management of risks associated with scrapie and prion-related diseases. According to the latest EFSA report (2022), a total of 224 cases of scrapie were reported in the EU-27 and Northern Ireland [16]. Among these, 216 cases were classified as classical scrapie, constituting 96.4% of the total, with Cyprus representing 62% of classical scrapie cases. Additionally, eight cases were identified as having atypical scrapie, accounting for 3.6% of the reported cases.

The *PRNP* gene, which encodes the PrP^C^ protein, plays a crucial role in determining susceptibility and resistance to scrapie [17]. Several genetic variations have been identified in goat populations [18–20]. Barillet’s study considered six amino acid substitutions (codons 127, 142, 146, 154, 211, and 222), which were found to confer some level of resistance to classical or atypical scrapie [21]. However, in the latest EFSA report, only the K222, D146, and S146 alleles were identified as offering significant protection against classical scrapie strains commonly observed in the European Union goat population. In particular, the D146 and S146 alleles provide a comparable level of resistance [16, 21]. Various studies have demonstrated that specific polymorphisms, such as S127 and M142, extend the incubation period of the disease but confer less resistance than do codons 146 and 222 [22–25]. On the other hand, H154 has been identified as a potential risk factor for atypical scrapie [26]. Furthermore, previous studies, such as that conducted by Cinar et al. in 2018, confirmed that goats with specific alleles (S/D146 and K222) exhibited strong resistance [27]. The protective *PRNP* alleles K222 and S/D146 are more prevalent in certain local goat breeds and specific geographic regions. For instance, Garganica Breed (Southern Italy) has a greater frequency of K222 [28]; Small East African Breed (Tanzania) has a greater frequency of S/D146 [29]; Damascus-related Breeds (Cyprus and Turkey) have greater frequencies of K222 or S/D146 [30, 31]; and Boer Goat (Netherlands, UK, and USA) has a greater frequency of the D146 allele [22, 32, 33]. Surveillance plans based on the *PRNP* genotype are crucial for disease prevention and promote the long-term sustainability and prosperity of goat farms. The plan involves genotyping of *PRNP* at positions corresponding to codons 146 and 222 to assess resistance or susceptibility to classical scrapie. While targeted selection programs are not in place, the monitoring of *PRNP* genotypes helps in managing preventive and control measures for scrapie.

In Italy, the surveillance of scrapie in goats is part of an annual program that includes both active and passive monitoring. Active surveillance involves conducting 10 000 rapid tests for each species (sheep and goats), whereas passive surveillance relies on the reporting and subsequent confirmation of suspected cases. Additionally, this surveillance program has initiated the identification of carriers of the K222 allele and the establishment of a national database of goats resistant or semi-resistant to scrapie, as outlined in the Ministry of Health’s note No. 19770–18/07/2019 [34]. Genotyping-based surveillance helps in implementing effective scrapie prevention measures, guiding regulatory decisions, and informing future breeding programs aimed at enhancing resistance to this disease. The guidelines for the control of TSE in sheep and goat herds in Italy were recently updated (2023) [35].

Goat farming in Italy represents a significant component of the national livestock landscape. With a population exceeding one million goats spread across the country, notable contributions come from southern regions such as Sicily, Calabria, and Apulia. However, a significant presence is observed even in northern regions such as Lombardy, with nearly 90 000 goats. The diversity of goat breeds raised in Lombardy is notable, reflecting the richness and heterogeneity of the region’s livestock heritage. Alpine goats, accounting for 22.7%, stand out as the most widespread breed, followed by the Saanen breed and other crossbreeds and selectively bred varieties, each with unique characteristics that reflect the history and needs of local farming.

The present study aimed to assess the frequency of *PRNP* gene polymorphisms at codons 127, 142, 146, 154, 211, 222, and 240 in goats from four farms in the Lombardy region in Italy. This study aimed to provide an updated overview of the *PRNP* genotype distribution in Lombardy, considering cosmopolitan breeds such as Saanen and Alpine and including crossbreeds in the analysis.

## Materials and methods

### Sample collection and genotyping

A total of 956 apparently healthy animals were sampled from four distinct farms within the Lombardy region of Italy using ear tissue sampling units (TSU) for adult individuals and bioptic ear tags for newborn goats. These four farms were chosen for several reasons, including their representativity in terms of diverse goat farming practices and herd sizes within the Lombardy region. Additionally, all of these farmers use both artificial insemination and natural mating: one of them buys some of its breeding bucks from another farm located in the Lombardy region, while the others also use bucks that are born on their own farm. This diversity in breeding practices provides valuable insights into the genetic variability within the region's goat populations. To ensure a representative sample, the selected farms are geographically spread across Lombardy and lack direct relationships with one another. The sample sizes for each farm were as follows: Flock 1 (n. 117), Flock 2 (n. 83), Flock 3 (n. 456), and Flock 4 (n. 300). The range of farm sizes and breeding methods reflects the overall landscape of goat farming in Lombardy.

A Quick-DNA^™^ Miniprep kit (Zymo Research) was used to extract DNA from the ear tissue according to the provided protocol. All the collected samples were classified in a project structured database and stored at the University of Milan tissue repository Animal Bio-Arkivi [36].

The sampled animals consisted of two distinct breeds, Alpine (n. 692) and Saanen (n. 168) goats, along with 96 cross-bred goats. This last population, identified as “mixed”, incorporates different genetic contributions from both Alpine and Saanen goats and from Dutch-origin bucks used for artificial insemination.

This study is part of the CapraGEN project (Applicazione della GENomica negli Allevamenti di Capra da Latte) funded by the Lombardy Region. All animals collected for this research were genotyped with the Neogen GGP Goat 70 k chip, which consists of approximately 70 000 single nucleotide polymorphisms (SNPs). The chip also included markers located within and in proximity to the *PRNP* gene. Initially, we delineated the genomic segment corresponding to the *PRNP* gene, including its upstream and downstream regions. Within this defined region, the genotyping chip contains 18 distinct SNPs, as detailed in Additional file 1, where each position is reported along with their respective Ensembl SNP IDs, if available [37]. Among these SNPs, four and five were located in the upstream and downstream regions, respectively, while nine were located within the coding region of the *PRNP* gene. Of these nine SNPs, two have been categorized as synonymous variants, and consequently, allele frequencies have not been examined. The remaining seven positions have undergone further investigation because they correspond to missense variants, and they are valuable for identifying potential mutated alleles associated with scrapie resistance according to available references (Additional file 1). The SNPs coordinates are in concordance with those of the ARS1.2 goat genome.

### Statistical analysis

All comparative analyses were conducted across the three distinct populations.

Seven positions within the coding region of the *PRNP* gene were used for the analysis. Mutations in these positions, corresponding to codons 127, 142, 146, 154, 211, 222, and 240, result in amino acid substitutions. The genotyping results correspond to the detection of the two alleles at each analysed position for every individual. The combination of these seven positions results in the formation of a haplotype. The presence of loci with double heterozygosity poses challenges in determining the precise allelic phases needed for reconstructing haplotypes. To address this challenge, we computed maximum likelihood estimates of haplotypes by taking into account the frequencies of allelic variants within the analysed codons. We employed the expectation–maximization (EM) method implemented in Arlequin 3.5.2 under the assumptions of Hardy–Weinberg equilibrium and an unknown gametic phase, as described by Barillet et al. [21, 38, 39].

This algorithm takes the two pseudo-haplotypes as input for each sample and subsequently generates estimates for the haplotypes along with their frequencies in the sampled population.

On the basis of haplotype reconstruction frequencies, the EM method provides estimates for the likelihood of each genotype being present in the population.

The variant allele and haplotype frequencies estimated in the three populations were compared using ${\chi }^{2}$ tests.

Linkage disequilibrium was calculated from the reconstructed haplotypes in Arlequin 3.5.2 under the hypothesis that the gametic phase is not known [38, 39]. The frequencies of polymorphisms identified at each position, as well as the frequencies of haplotypes and their genotypes, were compared with those reported in previous studies.

## Results

### Goat *PRNP* polymorphisms: allele and genotype frequencies at a single locus

Seven missense variants within the coding region of *PRNP* were genotyped (the mean call rate was 98.1%), and all these positions were associated with amino acid substitutions. Amino acids are cited according to the IUPAC nomenclature with one conventional letter code [40]. The seven examined codons are presented as follows: the first letter represents the reference amino acid, and the second letter denotes the alternative variant (G127S, I142M, N146S, R154H, R211Q, Q222K, or S240P). In Additional file 1, the information for each genomic allele and the corresponding amino acid variant is reported for the investigated positions.

**Figure 1:**
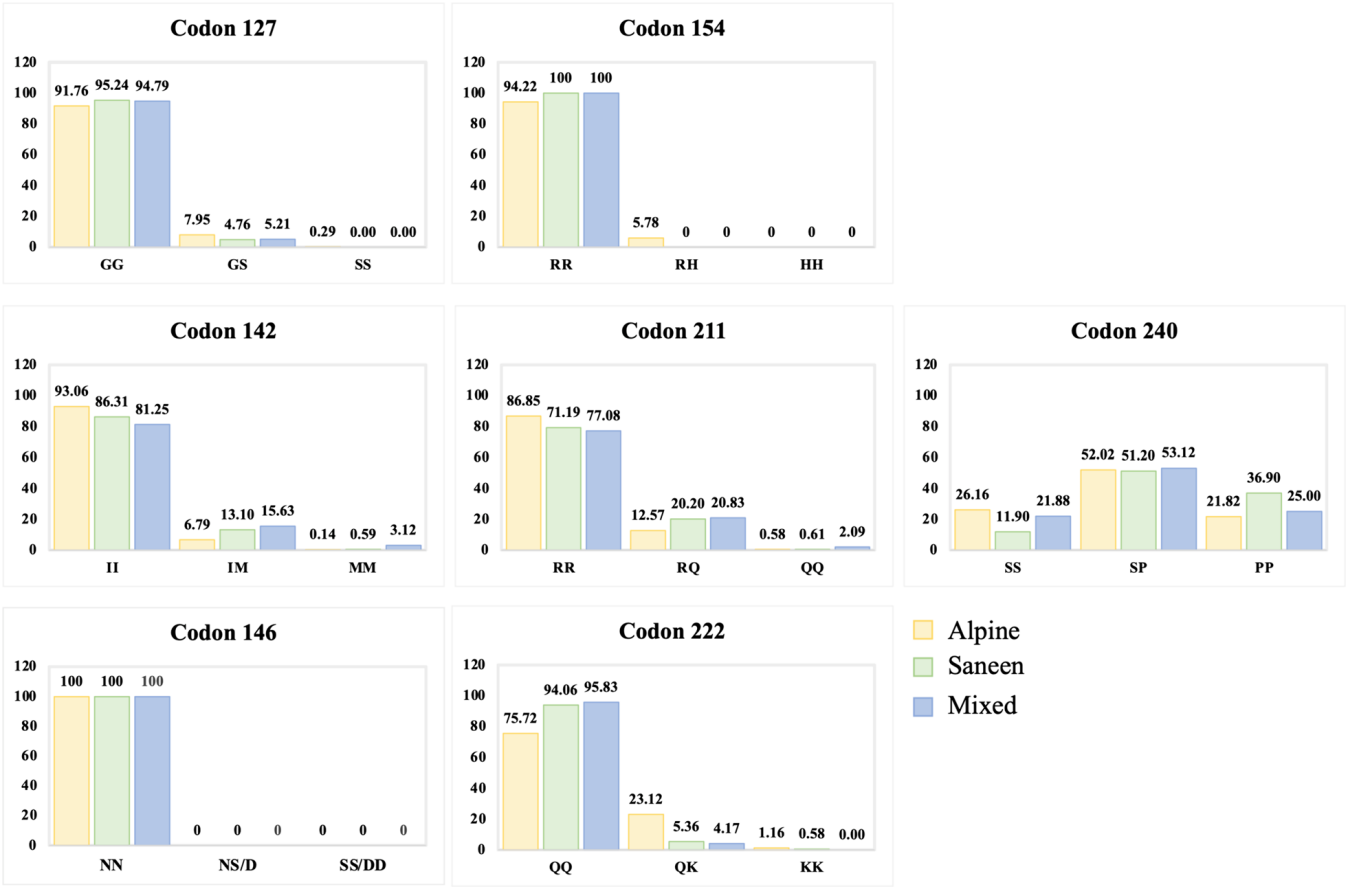
**Genotypes of** ***PRNP* variants calculated for the seven codons in the Alpine, Saneen, and mixed goat populations.**

Figure 1 illustrates the genotype frequencies, while Table 1 reports the allele frequencies at each position within each population.

**Table 1:** **Allele frequencies of** ***PRNP*** **polymorphisms at codons 127, 142, 146, 154, 211, 222 and 240 in the studied populations**

Codon position	aa	Frequency				χ^2^	P-value
		Alpine	Saanen	mixed	on Total		
		n. 692	n. 168	n. 96	n. 956		
127	G	0.957	0.976	0.974	0.962	0.094	0.760
	S	0.043	0.024	0.026	0.038		
142	I	0.965	0.929	0.891	0.951	1.552	0.213
	M	0.035	0.071	0.109	0.049		
146	N	1	1	1	1	–	–
	S/D	–	–	–	–		
154	R	0.971	1	1	0.979	0.258	0.612
	H	0.029	–	–	0.021		
211	R	0.931	0.893	0.875	0.919	0.363	0.547
	Q	0.069	0.107	0.125	0.081		
222	Q	0.873	0.967	0.979	0.900	1.037	0.308
	K	0.127	0.033	0.021	0.100		
240	S	0.522	0.375	0.484	0.492	2.770	0.096
	P	0.478	0.625	0.516	0.508		

There were few amino acid substitutions in the three populations (2.1–12.7%), with an average substitution of 11.4%. The only exception is represented by codon 240, with an average substitution frequency of 50.8%.

Substitutions were detected in all positions in the Alpine, Saanen or mixed breeds, except for position 146, where all animals exhibited the reference variant corresponding to asparagine.

In addition, the Saanen and mixed population lack the alternative allele at codon 154.

No significant differences between populations were evident. At codon 240, the Alpine breed had a frequency of 0.478 for the P240 variant, the Saanen breed had a frequency of 0.625, and the frequency of mixed goats had an intermediate value of 0.516; however, no significant differences were identified (*p* value of 0.096).

Regarding genotypic frequency, it can be observed from Figure 1 that the majority of mutated alleles are present in the population in the heterozygous form, ranging from 4.17 to 53.12%. The highest value is reached in codon 240, where the heterozygous form of SP240 is the most represented.

Homozygous mutated genotypes reached the highest frequencies in the mixed breed population, exhibiting frequencies of 3.12 and 2.09% for the MM142 and QQ211 genotypes, respectively.

The homozygous reference genotypes ranged from 11.90% at position 240–100% at position 146 and were the most represented, except at position 240.

### Haplotypes of the goat *PRNP* gene in Alpine, Saanen and mixed breed goats

The results obtained by generating a haplotype in 7 positions of the *PRNP* goat gene open reading frame (ORF) using the maximum-likelihood estimation method implemented in Arlequin 3.5.2 revealed the potential existence of 10 distinct haplotypes across the three populations. The haplotype frequencies are shown in Table 2. Only haplotypes with a frequency exceeding 1.00e-05 were considered for further analysis.

**Table 2:** **Haplotype frequencies for the goat** ***PRNP*** **gene at codons 127, 142, 146, 154, 211, 222, and 240 in 956 animals**

Haplotype	Codon							Frequency		
	127	142	146	154	211	222	240	Alpine	Saanen	mixed
1	G	I	N	R	R	Q	S	0.297	0.235	0.339
2	.	.	.	.	.	.	P	0.400	0.539	0.380
3	.	.	.	.	.	K	.	0.127	0.031	0.021
4	.	M	.	.	.	.	P	0.035	0.067	0.109
5	S	.	.	.	.	.	P	0.043	0.026	0.014
6	.	.	.	.	Q	.	.	0.069	0.125	0.104
7	.	.	.	H	.	.	.	0.029	–	–
8	S	.	.	.	Q	.	.	–	0.003	–
9	S	.	.	.	.	K	.	–	0.002	–
10	S	M	.	.	.	.	P	–	0.004	–

Alpine (n. 692), Saanen (n. 168), mixed breed goats (n. 96).

The G_127_I_142_N_146_R_154_R_211_Q_222_S_240_ haplotype is the wild-type haplotype, reported as H1 in Table 2. Four mutated haplotypes, denoted H2, H3, H6 and H7, are generated by a single codon mutation from the wild type (GINRRQP_240_, GINRRK_222_S, GINRQ_211_QS, GINH_154_RQS). These haplotypes display varying frequencies across populations. The most prevalent haplotype in the three populations was the one with a P240 mutation, with a frequency of 0.400 in the Alpine population, 0.539 in the Saanen population and 0.380 in the mixed population. Differences are evident in the frequencies of H3 present in 176 Alpine goats, 10 Saanen goats, and 4 mixed breed goats. Conversely, H6 was more frequently observed in Saanen and mixed breed goats, with values of 0.125 and 0.104, respectively, compared to 0.069 in Alpine goats. Clearly, even if the relative frequency of H6 is greater, the number of Saanen and mixed breed goats sharing that haplotype is lower than the number of Alpine goats having the same haplotype due to disparities in sample size.

After conducting a ${\chi }^{2}$ square test, we confirmed that the observed differences in frequencies among the different species were not statistically significant. The results of the ${\chi }^{2}$ square test are presented in Additional file 2.

H4 is the result of two simultaneous mutations, M142 and P240, with frequencies of 0.035 in Alpine goats, 0.067 in Saanen goats and 0.109 in mixed goats. Notably, these two codons result in complete linkage disequilibrium (Additional file 3). Indeed, the association between a mutation at position 142 and a mutation at position 240 is not exclusive to H4; this pattern is also evident in H10.

Notably, not all the haplotypes were identified in all three populations. Specifically, H7 was present only in the Alpine population at a low frequency, while H8, H9 and H10 were exclusive to Saanen goats. Importantly, this reconstruction was performed using the EM method. Haplotypes with very low frequencies (H8, H9, and H10) may not accurately represent the actual haplotypes present in animals, and phased haplotypes could be different.

### Haplotype genotypes of the goat *PRNP* gene in alpine, Saanen and mixed breed goats

Table 3 shows the 26 most likely genotypes found in the Alpine, Saanen and mixed populations. In certain instances, the algorithm used to infer genotypes yields two combinations for each pair of pseudo-haplotypes provided as inputs. In such cases, only genotypes with a frequency exceeding 0.05 were selected as the assigned genotypes.

**Table 3:** **Genotypes of the** ***PRNP*** **gene in 692 Alpine, 168 Saanen and 96 mixed breed goats**

Genotype		Alpine	Saanen	Mixed
H1/H1	GINRRQS/GINRRQS	0.088	0.055	0.115
H1/H2	GINRRQS/GINRRQP_240_	0.238	0.254	0.257
H1/H3	GINRRQS/GINRRK_222_S	0.076	0.014	0.014
H1/H4	GINRRQS/GM_142_NRRQP_240_	0.021	0.032	0.074
H1/H5	GINRRQS/S_127_INRRQP_240_	0.025	0.007	–
H1/H6	GINRRQS/GINRQ_211_QS	0.041	0.049	0.085
H1/H7	GINRRQS/GINH_154_RQS	0.017	–	–
H2/H2	GINRRQP_240_/GINRRQP_240_	0.160	0.291	0.145
H2/H3	GINRRQP_240_/GINRRK_222_S	0.102	0.033	0.016
H2/H4	GINRRQP_240_/GM_142_NRRQP_240_	0.028	0.073	0.083
H2/H5	GINRRQP_240_/S_127_INRRQP_240_	0.034	0.015	0.020
H2/H6	GINRRQP_240_/GINRQ_211_QS	0.055	0.112	0.095
H2/H7	GINRRQP_240_/GINH_154_RQS	0.023	–	–
H3/H3	GINRRK_222_S/GINRRK_222_S	0.016	0.001	–
H3/H4	GINRRK_222_S/GM_142_NRRQP_240_	0.009	0.004	–
H3/H5	GINRRK_222_S/S_127_INRRQP_240_	0.011	0.003	–
H3/H6	GINRRK_222_S/GINRQ_211_QS	0.017	0.006	–
H3/H7	GINRRK_222_S/GINH_154_RQS	0.007	–	–
H4/H4	GM_142_NRRQP_240_/GM_142_NRRQP_240_	0.001	0.005	0.012
H4/H5	GM_142_NRRQP_240_/S_127_INRRQP_240_	0.003	0.006	–
H4/H6	GM_142_NRRQP_240_/GINRQ_211_QS	0.005	0.014	0.027
H5/H5	S_127_INRRQP_240_/S_127_INRRQP_240_	0.002	–	–
H5/H6	S_127_INRRQP_240_/GINRQ_211_QS	0.006	0.006	–
H5/H7	S_127_INRRQP_240_/GINH_154_RQS	0.002	–	–
H6/H6	GINRQ_211_QS/GINRQ_211_QS	0.005	0.011	0.016
H6/H7	GINRQ_211_QS/GINH_154_RQS	0.004	–	–

The prevailing genotype in both the Alpine and mixed-bred populations was GINRRQS/GINRRQP_240_, with frequencies of 0.238 and 0.257, respectively. In the Saanen population, the most frequent genotype is GINRRQP_240_/GINRRQP_240_, occurring at a frequency of 0.291.

Not all genotypes exhibited a uniform distribution across the populations, as evidenced by the outcomes derived from the comparison of polymorphism frequencies at individual positions, as presented in Table 1.

The genotype GINRRQP_240_/GINRRK_222_S, for instance, is prevalent in Alpine goats, with a frequency of 0.102, while it is observed at lower frequencies of 0.033 in Saanen goats and 0.016 in the mixed population. It is noteworthy that the high number of different genotypes in the Alpine breed could be attributed, in part, to the larger sample size in comparison to the other two breeds.

### Comparison with other studies

The outcomes of the comparisons among prior studies concentrating on the same breeds are presented in Table 4. When conducting the comparison, it is important to emphasize that prior studies have also examined polymorphisms in different positions. In our analysis, we specifically compared the haplotypes identified in other research studies with the positions we investigated in the current study. In addition, the statistical methods used to reconstruct haplotypes are different, except for those used by Barillet et al., who used the EM algorithm, as in this work [21]. In Torricelli et al., haplotypes were reconstructed using Phase software [41, 42].

**Table 4:** **Comparison of haplotype frequencies in Alpine and Saanen goats from previous studies**

Haplotype	This study		Torricelli 2021 [42]	Barillet 2009 [21]		Acutis 2008 [28]		Acutis 2006 [43]
	Alpine (n. 692)	Saanen (n. 168)	Saanen (n. 19)	Saanen (n.184)	Alpine (n.220)	Alpine (n. 84)	Saanen (n. 69)	Alpine (n. 177*)
H1	0.297	0.339	0.263	0.212	0.350	0.298	0.188	0.218
H2	0.400	0.380	0.368	0.451	0.314	0.327	0.587	0.396
H3	0.127	0.021	0.026	0.049	0.074	0.024	0.030	0.071
H4	0.035	0.067	0.053	0.087	0.039	0.089	0.057	0.020
H5	0.043	0.026	–	0.011	0.098	0.012	0.021	0.011
H6	0.069	0.125	0.237	0.185	0.071	0.137	0.102	–
H7	0.029	–	–	0.005	0.054	0.113	–	0.085

() Number of analysed samples per breed: *frequencies refer to Alpine and Maltese goats analysed together in this study.

The two remaining studies [28, 42] were based on sequencing technologies that included phased genotypes as outcomes.

The predominant haplotype identified in earlier research studies was H2, characterized by the P240 mutation. The frequencies observed for this haplotype in our populations align with values reported in prior studies, ranging from 0.314 in the Alpine population to 0.587 in the Saanen population, as documented by Acutis et al. [28].

The haplotypes detected in our populations are consistent with findings from previous studies. Notably, H7, characterized by the H154 mutation and exclusively present in Alpine goats in our population, is absent in other Saanen populations, except for a study by Barillet et al., where this haplotype was identified in the Saanen population but with a very low frequency of 0.005 [21].

## Discussion

In summary, while the scrapie prevalence this study provides a general overview of the frequency of animals carrying scrapie resistance alleles in four goat farms in the Lombardy region, each with distinct breeding practices. Notably, no scrapie cases were identified among the goats on these farms, making this an ideal setting for studying the distribution of resistance-related polymorphisms. By identifying the presence of these alleles, we could guide preventive measures and support effective breeding strategies. Additionally, early detection of susceptible animals will allow farmers to make informed decisions regarding management and, if necessary, the isolation of affected individuals.

In this study, polymorphisms of seven SNPs mapped within the ORF of the *PRNP* gene were investigated in 956 goats farmed from 4 different herds, and haplotypes were reconstructed using the EM method.

Regarding the two main codons conferring resistance to scrapie according to the European Union legislation, no allele (S/D) with a strong protective effect was identified at position 146 (100% NN146), while a low allele frequency of K at position 222 (total value of 10%) was observed.

The protective effect of K222 mutation against classical scrapie has also been demonstrated by Acutis et al. and Vaccari et al. [43, 44].

The protective allele is present in the studied population mainly in heterozygous forms (genotype frequency ranging from 4.17 to 23.12%). Only a few homozygous animals (KK222) were identified, and these animals were all females belonging to both the Alpine (ranging from 1 in Flock 2–7 animals in Flock 4) and Saanen (only 1 in Flock 3) breeds.

The low frequency of the homozygous form could be attributed to the absence of a specific selection strategy in the four farms analysed, with the exception of one farm participating in a regional surveillance plan, which included genotyping of all males and the newborn bucks of the *PRNP* gene at positions 146 and 222.

Concerning mutations in the other analysed positions, the S127 allele, found in 72 animals across the three populations, is known to be able to extend the incubation time of TSE, as reported by Goldmann, 2011 [22]. In contrast, Barillet et al. reported that G127S does not have any effect, and they considered only mutations at other positions [21]. However, it is important to note that this mutation is present in only a limited proportion of the studied populations.

Moreover, the M142 mutation, found in 94 animals, has previously been identified as a factor influencing the incubation period of scrapie prions in goats, as reported by Goldmann et al. [23].

Barillet et al. identified linkage disequilibrium between alleles M142 and P240, a finding consistent with our results. Furthermore, they revealed that the genotypes IM_142_ and PP_240_, characterized by heterozygosity in the first position and homozygosity in the second position, confer a protective effect. This genotype corresponds to the H2/H4 genotype in the present study, with an average frequency of 0.061 across three populations in our research.

Mutation H154, found only in 40 Alpine goats, has been previously associated with a delayed progression of the disease, known as a risk factor for atypical scrapie.

The H154R genotype is also present in sheep, where it has been described as protective against classical scrapie infection [17, 45]. Nevertheless, the H154R genotype is also known to be a risk factor for atypical scrapie in sheep [46]. In addition, the Q211 polymorphism, which seems to prolong the incubation time, has been detected in 155 animals [28]. According to their analysis, Barillet et al. reported that animals heterozygous for the RH154, RQ211, and QK222 positions were significantly less susceptible to infection [21].

In the present study, these heterozygous animals were associated with genotypes H1/H7, H1/H6, and H1/H3, respectively.

Barillet et al. observed 6 different haplotypes in affected goats. The affected goats presented the wild-type haplotype H1 and five mutated haplotypes, H2, H3, H4, H6 and H7, which were also found in our populations.

Fortunately, none of the animals analysed exhibited symptoms associated with the disease. However, the conclusions drawn by Barillet et al. once again underscore the critical importance of implementing a selection strategy aimed at mitigating the spread of the disease associated with these potentially harmful haplotypes.

The varied breeding practices in the Lombardy region, such as the widespread use of artificial insemination and the sourcing of breeding bucks from other farms, could affect the genetic variability of the *PRNP* alleles. This diversity could lead to greater genetic variability and a broader range of resistance traits, which might explain the observed distribution of *PRNP* alleles in the four studied farms.

Understanding the variability across the seven positions not only in male goats but also in the whole flock is crucial for establishing breeding plans within the farm. Furthermore, it presents another advantage linked to the widespread practice of summer pasturing, commonly adopted by farms situated in mountainous regions. Summer pasturing facilitates encounters between animals of different species from different farms. While this practice is not established in the farms analysed, it is a diffuse practice in Italy. Knowing the resistance of the animals sold by a farm through genotyping could offer the advantage of introducing resistance to other farms, thereby protecting their animals during summer pasturing and preventing cross-species transmission.

This study highlights the variability in 7 positions associated with amino acid substitutions within the ORF of the *PRNP* gene in three populations of goats bred in the Lombardy region.

The variability in these codons, particularly concerning the K222 allele, represents a potential focal point for the implementation of a scrapie eradication breeding program, in accordance with the current Regulation EU 772/2020.

Nevertheless, the significance of the S/D146 allele in the eradication strategy has been emphasized; however, its absence in any of the 956 animals represents a limitation. The validation of the effects of all other codon mutations in healthy and affected animals will be a future goal.

The implementation of genotyping and other management strategies for scrapie in goats allows producers to take proactive measures to prevent the spread of the disease and maintain the health and welfare of their animals. In summary, while the scrapie prevalence in goats may be lower than that in sheep, the importance of monitoring and managing the disease remains significant due to the potential for economic losses, public health concerns, and the risk of cross-species transmission. Surveillance programs could be tailored to involve more rigorous genotyping and continuous monitoring to track the spread of resistance alleles among goat populations. This can guide breeding decisions, ensuring that resistance is maintained or increased over time. Effective scrapie prevention requires educating farmers about the significance of resistance alleles and their role in disease prevention. Outreach programs can raise awareness about the benefits of selective breeding and genotyping, encouraging farmers to participate in surveillance programs and adopt best practices to manage scrapie risk.

### Supplementary Information

Additional file 1. ***PRNP *variant IDs are reported along with chromosomal positions on the ARS UCD 1.2 genome, reference and alternative alleles, corresponding amino acids, genomic locations, variant types, and associated bibliographic references.**

Additional file 2. **Results of the **${\chi }^{2}$**square test for haplotype frequencies in the alpine, Saanen and mixed populations.**

Additional file 3. **Results of linkage disequilibrium analysis for each population.**

Additional file 4. **Genotype frequencies at each locus calculated for the four flocks.**

## Data Availability

All the data generated and analysed during this study are included in this published article and its supplementary information files.

## Abbreviations

*PRNP*: prion protein-encoding gene
EC: European Commission
TSE: transmissible spongiform encephalopathies
BSE: bovine spongiform encephalopathy
CWD: Chronic wasting disease
CNS: Central nervous system
PrP^C^: cellular form of the prion protein
PrP^Sc^: prion protein associated with scrapie
EFSA: European food safety authority
TSU: tissue sampling unit
ORF: open reading frame
EM: expectation–maximization
