# Supplementary figures and images for: Exploring the genetic variability of the PRNP gene at codons 127, 142, 146, 154, 211, 222, and 240 in goats farmed in the Lombardy Region, Italy
Source: Vet Res. 2024 Aug 6;55:99. doi: 10.1186/s13567-024-01353-3 (PMC11304840; doi:10.1186/s13567-024-01353-3)

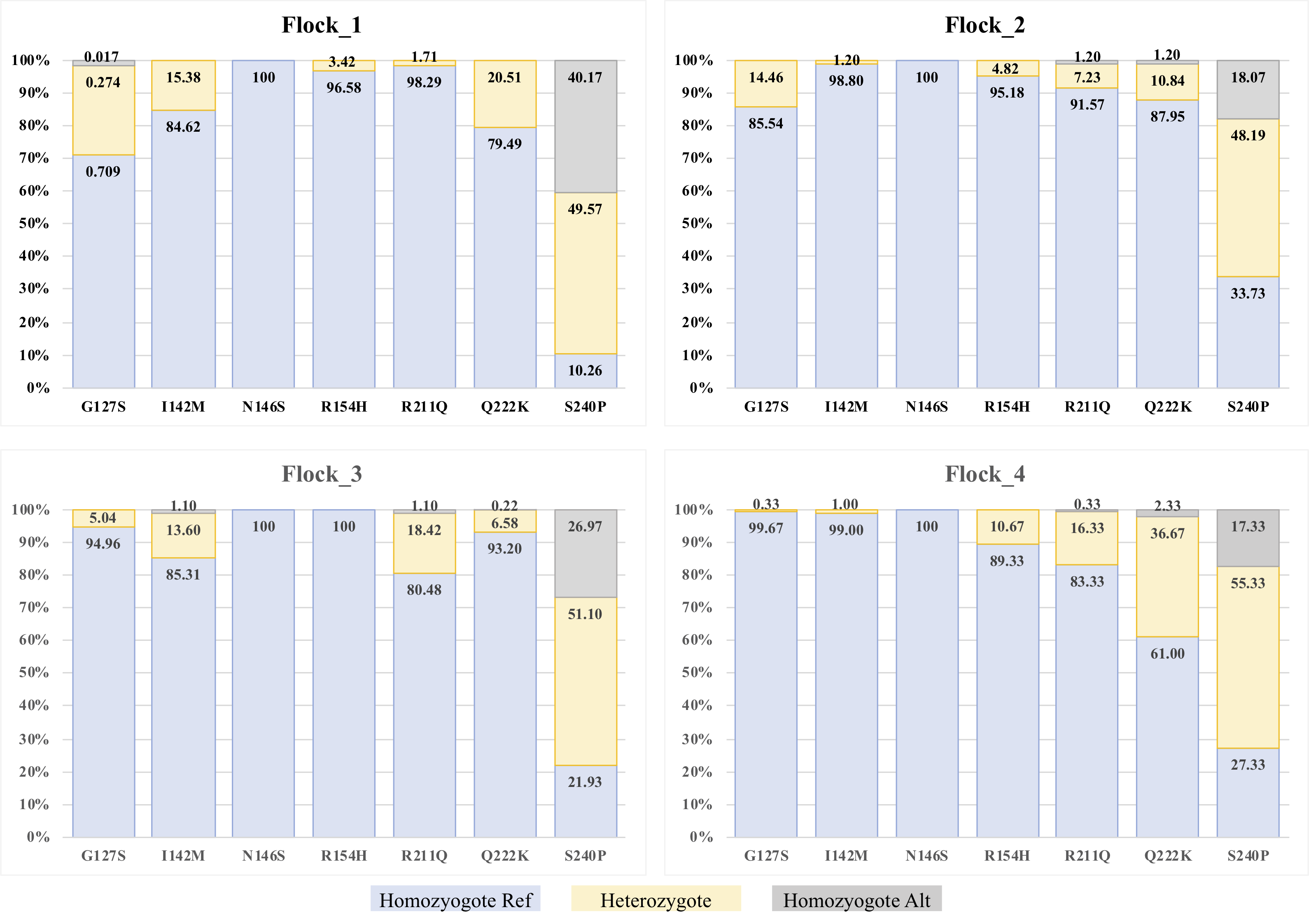

Supplement: Supplementary file 4 — Additional file 4. Genotype frequencies at each locus calculated for the four flocks. [file 13567_2024_1353_MOESM4_ESM.tiff]
